# Detection of ON1 and novel genotypes of human respiratory syncytial virus and emergence of palivizumab resistance in Lebanon

**DOI:** 10.1371/journal.pone.0212687

**Published:** 2019-02-21

**Authors:** Hadi Abou-El-Hassan, Elie Massaad, Nadia Soudani, Aia Assaf-Casals, Rouba Shaker, Mireille Lteif Khoury, Soha Ghanem, Maria Karam, Rabih Andary, Reiko Saito, Ghassan Dbaibo, Hassan Zaraket

**Affiliations:** 1 Department of Experimental Pathology, Immunology, and Microbiology, Faculty of Medicine, American University of Beirut, Beirut, Lebanon; 2 Center for Infectious Diseases Research, American University of Beirut, Beirut, Lebanon; 3 Department of Biology, Faculty of Sciences, EDST, Lebanese University, Hadath, Lebanon; 4 Department of Pediatrics and Adolescent Medicine, Faculty of Medicine, American University of Beirut Medical Center, Beirut, Lebanon; 5 Department of Pediatrics, Makassed General Hospital, Beirut, Lebanon; 6 Kiserwan Medical Center, Jounieh, Lebanon; 7 Al-Jabal Hospital, Aley, Lebanon; 8 Division of International Health (Public Health), Graduate School of Medical and Dental Sciences, Niigata University, Niigata, Japan; 9 Department of Biochemistry and Molecular Genetics, Faculty of Medicine, American University of Beirut, Beirut, Lebanon; St. Jude Children's Research Hospital, UNITED STATES

## Abstract

Respiratory syncytial virus (RSV) is a common cause of respiratory tract infections in children and immunocompromised individuals. A multi-center surveillance of the epidemiologic and molecular characteristics of RSV circulating in Lebanon was performed. The attachment (G) and fusion (F) glycoproteins were analyzed and compared to those reported regionally and globally. 16% (83/519) of the nasopharyngeal swabs collected during the 2016/17 season tested positive for RSV; 50% (27/54) were RSV-A and 50% (27/54) were RSV-B. Phylogenetic analysis of the G glycoprotein revealed predominance of the RSVA ON1 genotype, in addition to two novel Lebanese genotype variants, hereby named LBA1 and LBA2, which descended from the ON1 and NA2 RSV-A genotypes, respectively. RSV-B strains belonged to BA9 genotype except for one BA10. Deduced amino acid sequences depicted several unique substitutions, alteration of glycosylation patterns and the emergence of palivizumab resistance among the Lebanese viruses. The emergence of ON1 and other novel genotypes that are resistant to palivizumab highlights the importance of monitoring RSV globally.

## Introduction

Respiratory syncytial virus (RSV) is a leading cause of morbidity and mortality in infants, young children and immunocompromised patients [1]. Nearly 70% of children are infected at least once with RSV in their first year and 100% by two years of age [2], making them at risk of developing pulmonary diseases later in life [3].

RSV belongs to the family *Paramyxoviridae*, order *Mononegavirales*. It is an enveloped virus containing a 15.2 kb linear, negative-sense single-stranded RNA genome that encodes 11 proteins. RSV is divided into two antigenic subgroups, A and B, based on the reactivity of the virus with monoclonal antibodies mainly against the attachment (G) and fusion (F) glycoproteins [4]. The G protein contains two hypervariable regions (HVR1/2) [5]. Based on the variability within HVR2, at least 13 RSV-A and 22 RSV-B genotypic clades have been identified [6]. RSV-A ON1 and NA1 and RSV-B BA are the major circulating genotypes worldwide [7–9]. The RSV-A ON1 genotype, with its characteristic 72-nucleotide duplication in HVR2, emerged during the 2010/11 season in Canada and spread to other countries [10].

The F glycoprotein belongs to type I viral fusion proteins, a class whose structure and function derives mostly from studies on influenza virus hemagglutinin [11], paramyxovirus [12] and HIV-1 envelope [13]. Upon infection, the metastable prefusion F protein undergoes remarkable structural rearrangements into a postfusion form of six chains (A, B, C, D, E, F) with preservation of neutralizing epitopes [14]. There are currently no vaccines approved for RSV, but several are currently in the preclinical and clinical trials development phases [15]. Palivizumab, a humanized monoclonal antibody that interacts with antigenic site II of the F protein, is available for prophylaxis in high risk infants [16]. Palivizumab-resistant variants have been reported but their global prevalence is not fully known [17, 18].

In this study, we investigated the demographic and clinical characteristics of circulating RSV, its genetic diversity and susceptibility to palivizumab.

## Materials and methods

### Study design

A multi-center surveillance study was conducted during the 2016/17 season (October to May). The sites included major referral hospitals and distributed across two major governorates (two each in Mount Lebanon and the Capital of Beirut) in Lebanon in which nearly 40% of the population resides. Members of the research team approached individuals who presented as out- or in-patients with fever (defined as >37.8°C) and cough with onset within the past 10 days. A written informed consent was secured from the research subjects themselves or their legal guardians if below 18 years of age before nasopharyngeal swabs (NPs) were collected. NPs were stored at 4°C for a maximum of 48 hours. RSV specimens detected by the rapid-detection kit QuickNavi Flu+RSV (Denka Seiken) during the 2014/15 and 2015/16 were included for sequence analysis purposes only. The study was approved by the Institutional Review Board of the American University of Beirut.

### RNA extraction and nucleic acid amplification reactions

Total RNA was extracted using PureLink Viral RNA/DNA Mini Kit (Invitrogen). All samples were initially screened by real-time RT-PCR using AgPath-ID One-Step kit (Applied Biosystems), with a primer/probe set specific for detecting the RSV G gene [19]. RT-PCR-positive samples were screened by conventional PCR to amplify HVR2 of the G gene [20]. The palivizumab target sequence of the F gene was amplified at an annealing temperature of 60°C using the following primers (5’-3’): RSV-A forward GTTCCAACAAAAGAACAACAGACT, RSV-A reverse AGTAACTTTGCTGTCTAACTATTT, RSV-B forward CAGCAGAAGAACAGCAGATTG and RSV-B reverse GAATAACTTTGTTGCCTTACTATCTG. Samples negative for RSV-A were screened for RSV-B.

### Sequencing and phylogenetic analysis

PCR products underwent Sanger sequencing at Macrogen Inc. (Seoul, South Korea). Sequences were aligned using CLUSTAL W, BioEdit 7.0 [21]. Phylogenetic trees were inferred using the maximum likelihood method by implementing the best fit nucleotide substitution model with 1000 bootstrap replicates using MEGA 7.0 [22]. Hasegawa-Kishino-Yano with Gamma distribution and Tamura-Nei with invariablility models were used as per the best fit model analysis for the RSV-A and RSV-B G gene sequences, respectively. A bootstrap value ≥70% and a pairwise (p) distance ≤0.07 was used as the criteria for designating a new genotype [23].

### Molecular analysis

A comprehensive search of GenBank RSV sequences was done and Lebanese sequences were assessed in relation to 9,265 RSV-A G gene, 4,347 RSV-B G gene, 1,952 RSV-A F gene and 933 RSV-B F gene sequences. Sequences were aligned using MAFFT [24]. Mutations were reported compared to the prototype strain. Mutations were indicated as unique if not previously reported at the same position in their corresponding genotypes. Unique mutations were identified using Unipro UGENE [25].

### Glycosylation analysis

The N- and O-glycosylation sites were predicted using NetNGlyc 1.0 [26] and NetOGlyc 4.0 [27], respectively, using a threshold value of 0.5.

### Computational analysis

Structures of the postfusion F glycoprotein (Protein Data Bank (PDB): 3RRT) and the Fab fragment of palivizumab (PDB: 2HWZ) were downloaded from PDB [14]. PyMOL v2.0 was used to introduce the mutations observed in our F amino acid sequences. Note that 3RRT as posted on PDB has the RSV-A Asn276 that was mutated in our RSV-B analysis to Ser276 to resemble the RSV-B wild-type F glycoprotein. F-bound palivizumab structures were predicted using Patchdock [28]. Polar contacts were identified using PyMOL and inter-chain interface interactions were analyzed using PDBsum [29].

### Statistical analysis

Chi-squared test was used to analyze the association of demographic and clinical variables with RSV infections. The descriptive and statistical analyses were performed using SPSS v24. Cases with missing or unreported data for a given variable were excluded from the analysis of that specific variable.

## Results

### Demographic and clinical characteristics

Demographic and clinical analyses were performed for the 2016/17 season, for which a complete dataset was available. During this season, 83/519 NPs (16%) tested positive for RSV (Table 1). Of these, 54.2% were males and the median age was 2 years (IQR 1–6). Children 2 years old and younger were most frequently affected by RSV (71.0%, p-value<0.001). Moderate fever (78.5%, p-value = 0.02), respiratory discomfort (43.4%, p-value = 0.024) and tachypnea (33.7%, p-value = 0.000) were more commonly exhibited by RSV-infected patients.

**Table 1:** Demographic and clinical characteristics of the 2016/17 season study participants.

		RSV Positive	RSV Negative	p-value
**Total**	**519**	**83 (16%)**	**436 (84%)**	
**Gender**				0.873
Male	285	45 (54.2%)	240 (55.2%)	
Female	233	38 (45.8%)	195 (44.8%)	
**Age Group (years)**				0.001
<2 (divided further below)	215	59 (71%)	156 (35.5%)	
*0–0.5*	*64*	*22 (26*.*5%)*	*42 (9*.*3%)*	
*0.51–1*	*60*	*17 (20*.*4%)*	*43 (9*.*9%)*	
*1.1–2*	*91*	*20 (24*.*1%)*	*71 (16*.*3%)*	
2.1–6	185	19 (22.9%)	166 (38.1%)	
6.1–12	55	1 (1.2%)	54 (12.4%)	
12.1–18	23	0 (0%)	23 (5.3%)	
18.1–60	25	2 (2.4%)	23 (5.3%)	
>60	16	2 (2.4%)	14 (3.2%)	
***Temperature at presentation***				0.02
37.2–37.8°C	4	1 (1.3%)	3 (0.7%)	
37.9–39.4°C	327	62 (78.5%)	265 (62.9%)	
>39.5°C	169	16 (20.3%)	153 (36.3%)	
**Cough** (inclusion criteria)				n/a
Yes	519	83	436	
**Headache**				0.399
Yes	86	5 (6.0%)	81 (18.6%)	
No	218	19 (22.9%)	199 (45.6%)	
**Sputum**				0.335
Yes	250	44 (53%)	206 (47.2%)	
No	269	39 (47%)	230 (52.8%)	
**Rhinorrhea**				0.669
Yes	462	75 (90.4%)	387 (88.8%)	
No	57	8 (9.6%)	49 (11.2%)	
**Sore throat**				0.039
Yes	65	4 (6.3%)	61 (16.4%)	
No	370	59 (93.7%)	311 (83.6%)	
**Tachypnea**				0.000
Yes	98	28 (33.7%)	70 (16.1%)	
No	419	55 (66.3%)	364 (83.9%)	
**Respiratory discomfort**^**a**^				0.024
Yes	169	36 (43.4%)	133 (30.6%)	
No	348	47 (56.6%)	301 (69.4%)	
**Abdominal symptoms**				0.064
Yes	86	8 (9.6%)	78 (17.9%)	
No	433	75 (90.4%)	358 (82.1%)	
**Abdominal pain**				0.070
Yes	71	2 (2.4%)	69 (15.8%)	
No	233	22 (26.5%)	211 (48.4%)	
**Nausea**				0.009
Yes	45	1 (1.2%)	44 (10.1%)	
No	473	81 (98.8%)	392 (89.9%)	
**Vomiting**				0.867
Yes	109	18 (21.7%)	91 (20.9%)	
No	410	65 (78.3%)	345 (79.1%)	
**Diarrhea**				0.570
Yes	137	24 (28.9%)	113 (25.9%)	
No	382	59 (71.1%)	323 (74.1%)	
**Malaise**				0.401
Yes	84	11 (13.3%)	73 (17%)	
No	429	72 (86.7%)	357 (83%)	
**Neurological symptoms**^**b**^				0.327
Yes	5	0 (0%)	5 (1.1%)	
No	514	83 (100%)	431 (98.9%)	
**Myalgia or arthralgia**				0.090
Yes	83	3 (3.6%)	80 (18.3%)	
No	221	21 (25.3%)	200 (45.9%)	
**Sick contacts**				0.802
Yes	190	31 (38.3%)	159 (36.8%)	
No	323	50 (61.7%)	273 (63.2%)	
**Surrounding cases**^**c**^				0.045
Yes	29	1 (3.7%)	28 (19.4%)	
No	142	26 (96.3%)	116 (80.6%)	
**International travel**				0.110
Yes	13	0 (0%)	13 (3%)	
No	504	83 (100%)	421 (97%)	
**Domestic travel**				0.582
Yes	25	5 (6%)	20 (4.6%)	
No	492	78 (94%)	414 (95.4%)	

^a^Respiratory discomfort includes dyspnea, retractions, accessory muscle use, grunting, nasal flaring, cyanosis and tachypnea

^b^Neurological symptoms include seizures, altered consciousness and coma

^c^Surrounding cases includes cases of acute respiratory infections at day care, school, home or work

**Fig 1:**
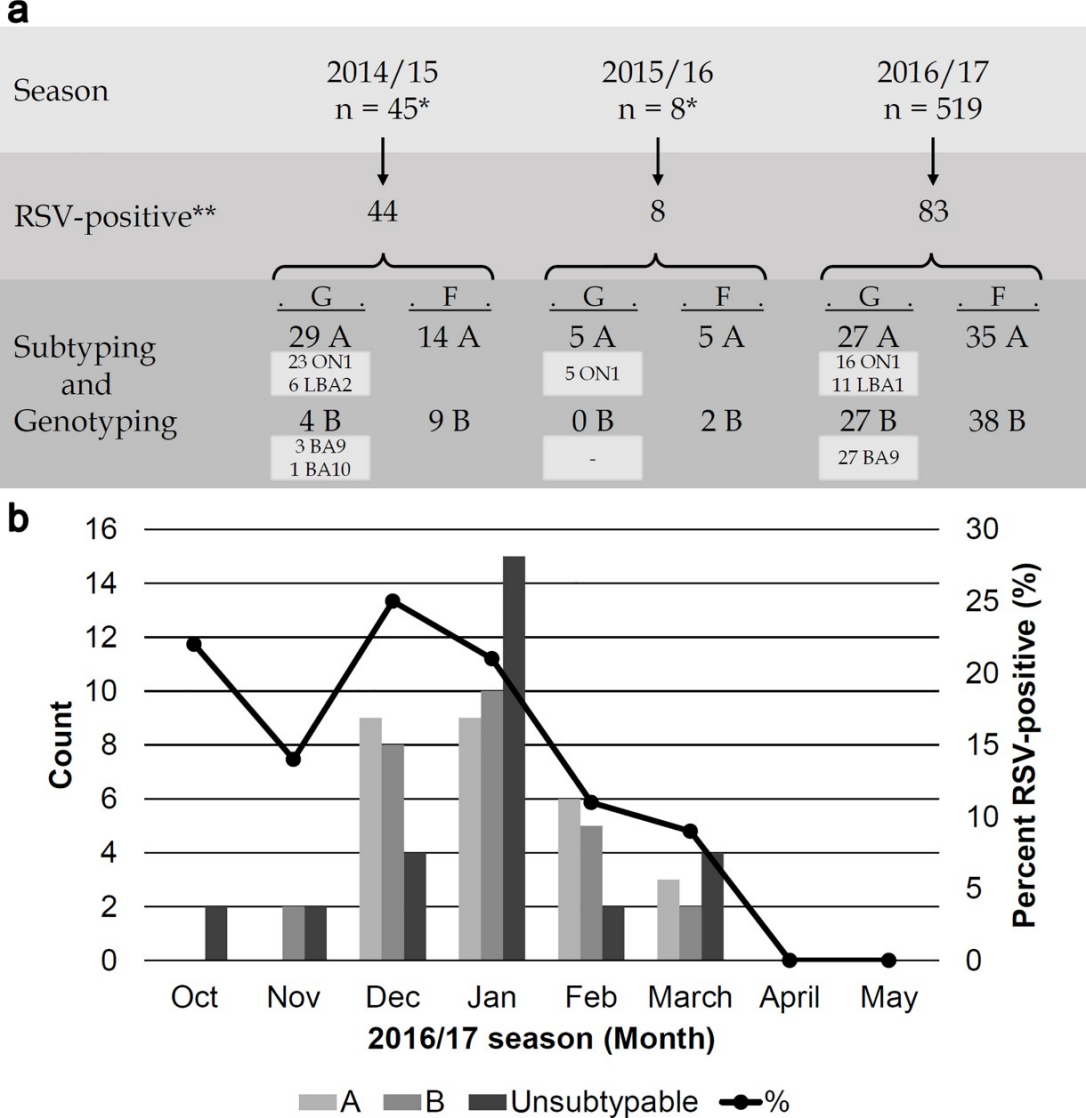
Distribution of RSV positive specimens. **(a)** Total count of RSV positive samples by season, subtype and genotype. *Indicates RSV-positive by a rapid-detection kit. **Indicates RSV-positive by real-time RT-PCR. **(b)** Monthly count of RSV positive samples by subtype and percent positive during the 2016/17 season. Samples for which G gene sequences could not be obtained were indicated as unsubtypable.

The G gene sequencing was attempted for all RSV-positive specimens that were identified in this study and was successful for 61 RSV-A and 31 RSV-B specimens. Molecular analysis revealed co-circulation of both RSV-A (50%, 27/54) and RSV-B (50%, 27/54) during the 2016/17 season (Fig 1A). RSV infections exhibited a clear seasonal trend and cases were detected October through March with a peak in December-January (Fig 1B).

### Novel RSV genotypic variants

**Fig 2:**
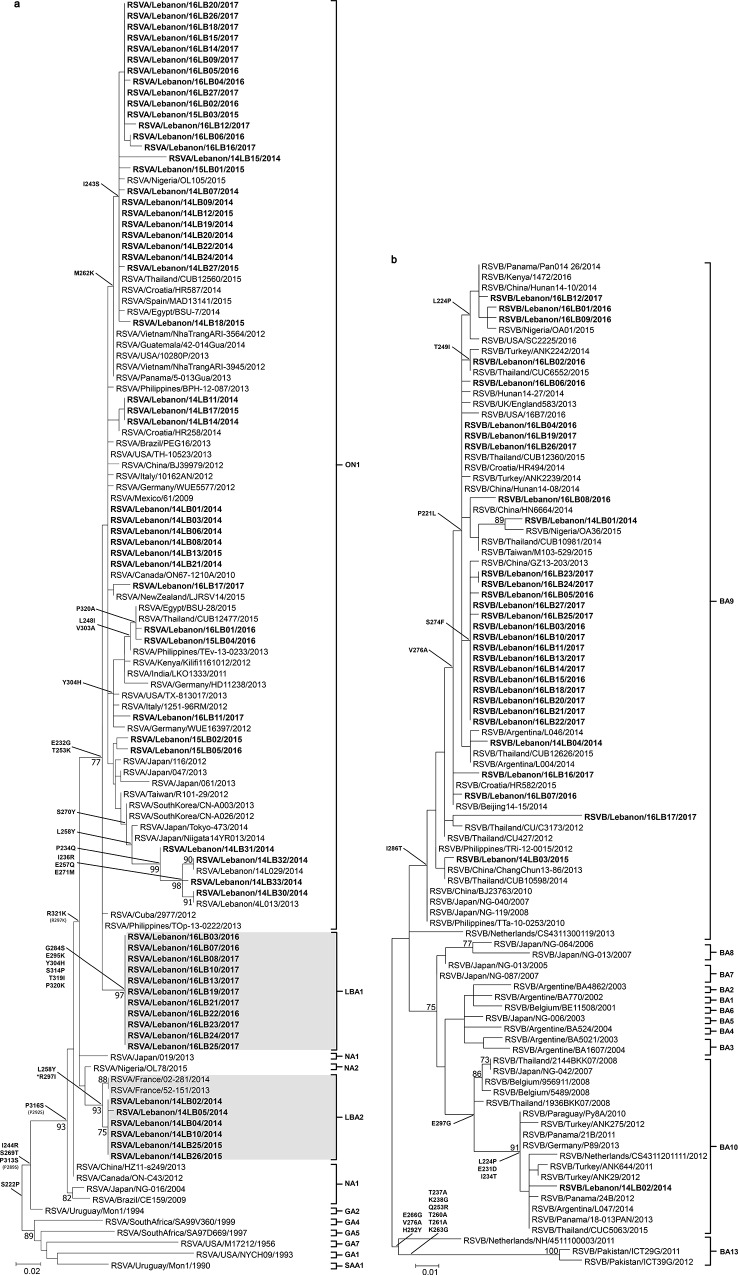
Phylogenetic trees of the Lebanese RSV-A and RSV-B specimens. **(a)** Phylogenetic analysis of the second hypervariable region of the G gene of the Lebanese RSV-A (bold) compared to reference strains. Novel LBA1 and LBA2 genotypes are highlighted. **(b)** Phylogenetic analysis of the second hypervariable region of the G gene of the Lebanese RSV-B (bold) compared to reference strains. Bootstrap values greater than 70 are shown at the branch nodes. The scale bars represent the number of nucleotide substitutions per site. Amino acid substitutions are shown for key tree nodes.*Indicates unique mutations in the Lebanese specimens. Nucleic acid sequences were deposited in GenBank under accession numbers MH687210—MH687270 for RSV-A G gene, MH687179—MH687209 for RSV-B G gene, MH687076—MH687129 for RSV-A F gene and MH687130—MH687178 for RSV-B F gene.

Phylogenetic analysis revealed that 44/61 of the Lebanese RSV-A specimens detected during the 2014/15, 2015/16 and 2016/17 seasons classified into the ON1 genotype (Fig 2A). A subset (n = 11) of the Lebanese RSV-A specimens from the 2016/17 season formed a unique cluster that branched off the ON1 lineage. This cluster had 97% bootstrap support and a p-distance < 0.001 meeting the criteria for designating a novel genotype [23] and was thus designated as LBA1. Another subset (n = 6) of Lebanese RSV-A specimens from the 2014/2015 season fell close to the NA2 genotype but formed a distinct cluster with a bootstrap value of 93% and p-distance ranging between 0.000 and 0.004, and was thus designated as LBA2. The RSV-A LBA2 cluster harbored two isolates from France that were reported in 2013 and 2014 [30].

The Lebanese RSV-B strains were classified as BA9 (n = 30) except for one specimen that belonged to BA10 (Fig 2B). The BA9 genotype was detected during the 2014/15 and the 2016/17 seasons. One specimen, RSVB/Lebanon/16LB17/2017, fell within BA9 but was characterized by a long branch indicating a relatively high number of substitutions.

### Detection of unique mutations and the emergence of palivizumab resistance

**Fig 3:**
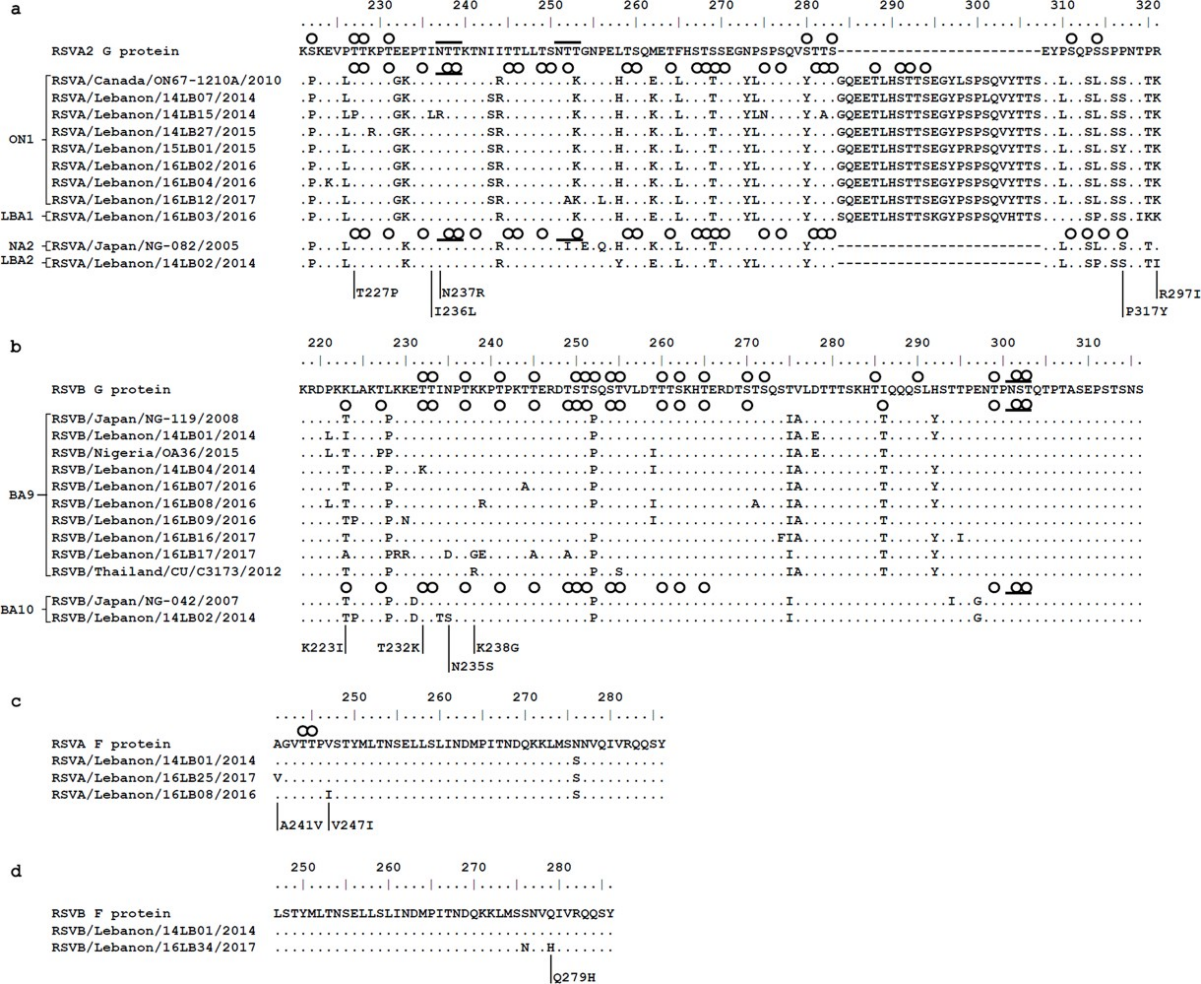
Amino acid sequence alignments of the G and F proteins. **(a)** Alignment of deduced amino acid sequences of the G protein of representative RSV-A strains relative to that of prototype strain A2 (RSVA2; M11486). A reference ON1 strain (RSVA/Canada/ON67-1210A/2010; JN257693) and a reference NA2 strain (RSVA/Japan/NG-082/2005; AB470479) were included for comparison. The alignment shown corresponds to positions 221 to 321 (297_-d_) of the second hypervariable region of RSV-A A2 strain G protein. **(b)** Alignment of deduced amino acid sequences of the G protein of representative RSV-B strains relative to that of the prototype BA4128/99B (RSVB; AY333364). A reference BA9 strain (RSVB/Nigeria/OA36/2015; HM459878) and a reference BA10 strain (RSVB/Japan/NG-042/2007; HM459884) were included for comparison. The alignment shown corresponds to positions 218 to 316 of the second hypervariable region of RSV-B strain BA4128/99B G protein. **(c)** Alignment of deduced amino acid sequences of the F protein of representative RSV-A strains relative to that of a prototype reference (RSVA/Australia/A2/1961; KJ155694). The alignment shown corresponds to positions 241 to 286 of the F protein. **(d)** Alignment of deduced amino acid sequences of the F protein of representative RSV-B strains relative to that of a prototype reference (D00334). The alignment shown corresponds to positions 247 to 286 of the F protein. Identical residues are identified as dots. Asn-Xaa-Ser/Thr sequons predicted to be N-glycosylated are highlighted with a line. Serine and threonine residues predicted to be O-glycosylated are highlighted with a circle. Unique amino acid substitutions are indicated.

To explain the distinct clustering of the novel RSV-A LBA1 and LBA2 strains, deduced amino acid sequences of representative ON1, LBA1 and LBA2 Lebanese specimens were aligned and examined relative to the region spanning residues 221–321 (221-297_-d_ [if duplication is removed]) of the prototype strain A2 G protein (M11486) and to globally reported strains including an ON1 (JN257693) and an NA2 (AB470479) reference strain (Fig 3A). Four unique substitutions were detected in the Lebanese ON1 specimens: T227P (n = 1), I236L (n = 1), N237R (n = 1) and P317Y (P293Y_-d_, n = 1). Specimens belonging to the LBA1 genotype (represented by 16LB03/2016) had 100% homology and shared six substitutions: G284S, E295K, Y304H, S314P (S290P_-d_), T319I (T295I_-d_) and P320K (P296K_-d_). Specimens of the novel LBA2 cluster had 100% homology and shared two substitutions: L258Y and R297I. This cluster carried the unique R297I mutation recently reported in two specimens (02-281/2014 and 52-151/2013) from France.

For the RSV-B specimens, the deduced amino acid sequences of representative BA9 and BA10 Lebanese strains were aligned and examined relative to the region spanning residues 218–316 of the prototype strain BA4128/99B (AY333364) and to globally reported strains including a BA9 (RSVB/Nigeria/OA36/2015; HM459878) and a BA10 (RSVB/Japan/NG-042/2007; HM459884) reference strain (Fig 3B). Three unique mutations were detected in the Lebanese BA9 specimens: K223I (n = 1), T232K (n = 1) and K238G (n = 1). The 16LB17/2017 strain was characterized by a relatively high number of substitutions: K223A, K229R, K230R, N235D, the unique K238G, K239E, T245A and T249A. Noteworthy, molecular analysis also revealed that the K238G mutation is only found in the RSV-B BA-13 genotype that has now appeared in BA9. The BA9 14LB01/2014 strain possessed a unique K223I substitution, in addition to a D278E substitution that was similar to Nigeria/OA36/2015, with which it clustered closely. For the Lebanese BA10 strain, one unique substitution (N235S) was detected.

To examine the susceptibility of our RSV-A specimens to palivizumab, the derived amino acid sequences (n = 54) were aligned relative to the region spanning residues 241–286 of the prototype RSV-A F protein (RSVA/Australia/A2/1961; KJ155694) (Fig 3C). Representative strain 14LB01/2014 is shown along with two specimens carrying distinct substitutions. Three substitutions were detected: two unique substitutions, A241V and V247I, in 16LB25/2017 and 16LB08/2016, respectively, and a previously reported N276S (n = 54) mutation. For RSV-B, derived amino acid sequences (n = 49) were aligned relative to the region spanning residues 247–286 of the prototype RSV-B F protein (D00334) (Fig 3D). Representative strain 14LB01/2014 is shown along with one specimen carrying distinct substitutions. Two amino acid substitutions were observed in 16LB34/2017: S276N and a unique Q279H. The RSV-A N276S mutation is associated with partial resistance to palivizumab [17, 31], unlike the RSV-B S276N mutation that does not affect palivizumab activity [32]. Substitutions A241V, V247I and Q279H have not been reported before and thus their effect on palivizumab neutralizing activity is unknown. Noteworthy, in Lebanon, palivizumab is only given to a small sub-population of infants with prematurity or congenital heart disease as per recommendations. Our study population was largely composed of otherwise healthy children, so it’s unlikely that a sizable number had received palivizumab.

### Substitutions N276S and Q279H in the F protein altered its binding to palivizumab

**Fig 4:**
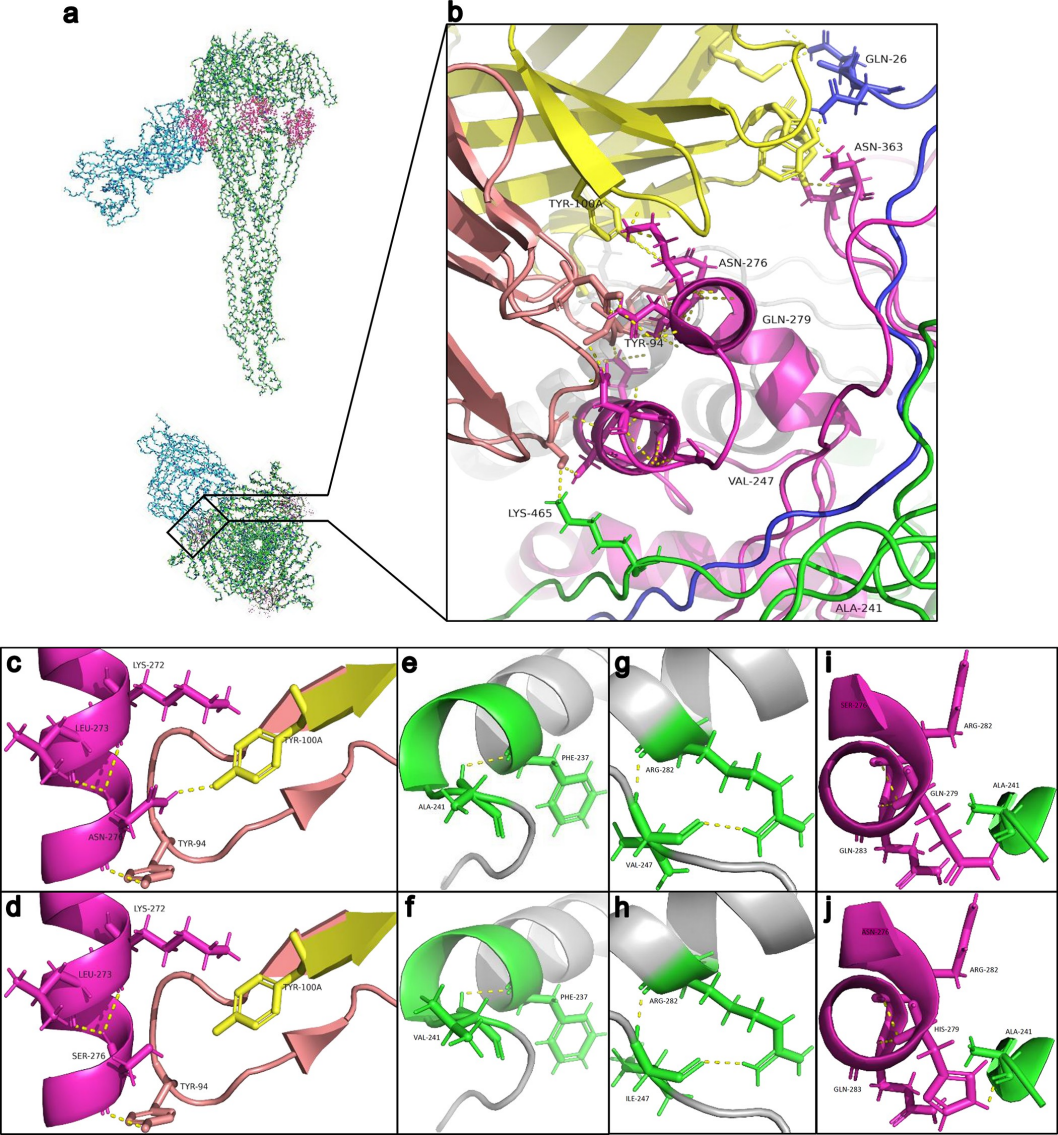
Structural elucidation of the interactions between F glycoprotein and palivizumab. **(a)** Predicted structure of the RSV-A postfusion F glycoprotein (PDB: 3RRT) bound to palivizumab Fab fragment (PBD: 2HWZ). Pink dots, antigenic site II (amino acid residues 258–275); upper panel, side view; lower panel, top view. **(b)** Predicted interface of polar inter-chain interactions between the RSV-A postfusion F glycoprotein antigenic site II and Fab fragment of palivizumab. The location of the four residues (Ala241, Val247, Asn276, Gln279) on the F glycoprotein for which mutations were observed in the Lebanese strains are shown. **(c)** Polar contacts by Asn276 in the RSV-A F glycoprotein vs **(d)** showing disappearance of a 2.3 Å polar bond with Tyr100 on Fab H chain due to the N276S substitution while maintaining the 2.9 Å polar bond with Tyr94. **(e)** Polar contacts by Ala241 in the RSV-A F glycoprotein vs **(f)** showing no changes of polar interactions with the A241V substitution. **(g)** Polar contacts by Val247 in the RSV-A F glycoprotein vs **(h)** showing no changes of polar interactions with the V247I substitution. **(i)** Polar contacts by Q279 in the RSV-B F glycoprotein vs **(j)** showing the appearance of a new 1.6 Å polar bond with Ala241 on an adjacent chain of the F glycoprotein due to the Q279H substitution. Yellow, palivizumab Fab heavy chain; orange, palivizumab Fab light chain; magneta, F glycoprotein F chain; blue, F glycoprotein E chain; green, F glycoprotein B chain; yellow dashed lines, polar contacts.

To further characterize the effect of the observed substitutions in the F glycoprotein on palivizumab binding, we analyzed the interactions between palivizumab and F glycoprotein carrying the mutations of interest (Fig 4). The observed mutations did not affect the predicted gross structure (Fig 4A and 4B) and did not change the PDBsum interface interactions (S1 Fig). Interacting polar bonds involved 14 residues: K465 in chain B, Q94 in chain C, K293 in chain D, Q26 and N27 in chain E, N254, S259, N262, N268, K272, S275, N276, S362 and N363 in chain F; assuming Fab binds antigenic site II of the F chain (Fig 4B).

Predicted RSV-A F glycoprotein structure carrying the N276S substitution bound to palivizumab revealed no structural changes but showed the loss of a 2.3 Å polar bond with Tyr100 of the palivizumab heavy chain while maintaining the 2.9 Å polar bond with Tyr94, although both asparagine and serine belong to the same class of polar amino acids (Fig 4C and 4D). Amino acids adjacent to the palivizumab binding site at positions 241 and 247 did not have any polar contacts with palivizumab. Thus, mutations at these residues are less likely to be implicated in resistance. In fact, A241V and V247I, which are close to the two O-glycosylated T244 and T245 residues, did not result in structural changes and maintained similar polar interactions as the wild-type residues (Fig 4E–4H). For the RSV-B F glycoprotein, the Q279H substitution in the F chain resulted in the formation of a new 1.6 Å polar contact with Ala241 in the B chain adding further stability to the F glycoprotein (Fig 4I and 4J). This increased stability is likely to enhance the susceptibility of this mutant to palivizumab.

### Distinct glycosylation patterns observed in the novel genotypes

Two N-glycosylation sites (Asn-Xaa-Ser/Thr where Xaa is any amino acid except proline) and 8 O-glycosylation sites (Ser or Thr) were predicted in the partial G protein sequence of the prototype strain A2 (Fig 3A). ON1 genotype (represented by Canada/ON67-1210A/2010) has 1 N-glycosylation site and 27 O-glycosylation sites. The Lebanese ON1 and LBA1 viruses had 1 N-glycosylation site which was lost in RSVA/Lebanon/14LB15/2014 due to the unique N237R mutation, and carried 30–40 O-glycosylation sites (Table 2). NA2 genotype (represented by Japan/NG-082/2005) has 2 N-glycosylation sites and 27 O-glycosylation sites, whereas the Lebanese LBA2 viruses have 30 N-glycosylation sites and 30 O-glycosylation sites.

**Table 2:** Representative Lebanese specimens with G protein substitutions and their predicted effect on glycosylation.

Genotype and Strain ID	Substitution	Effect on glycosylation	Number of O- glycosylations	Number of N- glycosylations
**RSV-A**				
**ON1**				
14LB07/2014	S301L**	– O-Glc	30	1
14LB15/2014	T227P*	– O-Glc	37	0
	I236L*	-		
	N237R*	– N-Glc		
	S275N	– O-Glc		
	T282A	– O-Glc		
14LB27/2015	K229R	-	40	1
15LB01/2015	S299R**	– O-Glc	31	1
	P317Y*	-		
16LB02/2016	G296S**	+ O-Glc	32	1
16LB04/2016	E224K	-	40	1
16LB12/2017	T252A	– O-Glc	39	1
	P256L	-		
**LBA1**				
16LB03/2016	N273Y	-	38	1
	P274L	-		
	G284S**	+ O-Glc		
	E295K**	-		
	Y304H**	-		
	S314P	– O-Glc		
	P317S	-		
	T319I	-		
	P320K	-		
**LBA2**				
14LB02/2014	L258Y	-	30	2
	R297I*	-		
**RSV-B**				
**BA9**				
14LB01/2014	K223I*	-	19	1
	D278E	-		
14LB04/2014	T232K*	– O-Glc	14	1
16LB07/2016	T244A	-	19	1
16LB08/2016	K239R	-	16	1
	T271A	-		
16LB09/2016	K230N	+ N-Glc	21	2
16LB16/2017	S274F	-	15	1
	T295I	-		
16LB17/2017	K223A	-	14	1
	K229R	-		
	K230R	-		
	N235D	-		
	K238G*	-		
	K239E	-		
	T245A	– O-Glc		
	T249A	– O-Glc		
**BA10**				
14LB02/2014	I234T	+ O-Glc	24	1
	N235S*	+ O-Glc		

– loss of N- or O-glycosylation (Glc) site.

+ gain of N- or O-Glc site.

- no change in glycosylation.

*unique mutations.

**mutation within the duplication.

**Fig 5:**
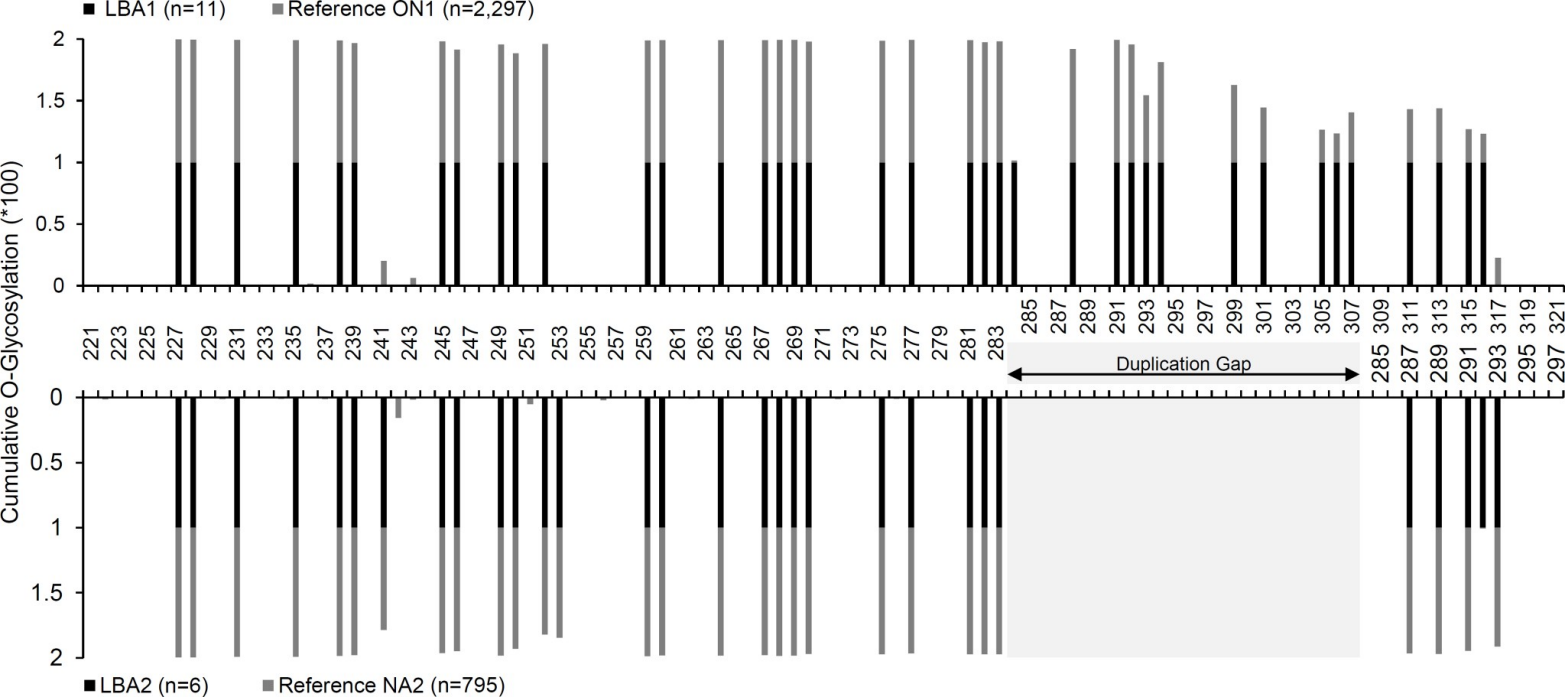
Glycosylation profiles of the Lebanese LBA1 and LBA2 specimens shown as cumulative proportion of glycosylated residues per RSV-A genotype. Highlighted region represents the duplication gap.

In order to determine whether characteristic O-glycosylation patterns exist for the novel LBA1 and LBA2 genotypes, sites of glycosylation in LBA1 were surveyed in all reported ON1 G sequences (n = 2,297) while sites of glycosylation in LBA2 were checked against NA2 G sequences (n = 795) (Fig 5). All positions predicted to be O-glycosylated in LBA1 were glycosylated in all ON1 sequences except at position 284 whose glycosylation appeared to be unique to LBA1 due to a conserved S284 that was retained from position 260 of the duplicated sequence. Also, LBA1 specimens uniformly carry O-glycosylated residues (T305, T306, S315, S316) which are infrequently possessed by the ON1 genotype. Likewise, all positions predicted to be O-glycosylated in LBA2 were glycosylated in NA2 sequences except position 292 whose glycosylation appeared to be unique to LBA2.

Regarding RSV-B, 1 N-glycosylation site and 20 O-glycosylation sites were predicted in the G protein of the prototype strain BA4128/99B (Fig 3B). BA9 genotype (represented by Japan/NG-119/2008) has 1 N-glycosylation site and 20 O-glycosylation sites. The Lebanese BA9 strains possessed 14–21 O-glycosylation sites and retained the N-glycosylation site present in the prototypic strain. One Lebanese specimen, 16LB09/2016, had an additional N-glycosylation due to a K230N substitution, and carried 21 O-glycosylation sites. BA10 genotype (represented by Japan/NG-042/2007) has 1 N-glycosylation site and 18 O-glycosylation sites whereas the Lebanese BA10 specimen had 1 N-glycosylation site and 24 O-glycosylation sites.

For the F protein amino acid sequences, two sites at positions 244 and 245 were predicted to be O-glycosylated in the RSV-A sequences whereas no N-glycosylation sites were predicted (Fig 3C). The previously reported N276S substitution did not yield a predicted O-glycosylation site. The V247I mutation in strain 16LB08/2016 resulted in the loss of a potential O-glycosylation site two residues upstream. Among the RSV-B F protein sequences, no sites were predicted to be N/O-glycosylated (Fig 3D).

## Discussion

In this study, RSV incidence among patients with acute respiratory infections during the 2016/17 season was 16% with a peak in December. Two epidemiologic studies of RSV were previously conducted in Lebanon; however, none attempted to genetically characterize the virus [33, 34]. Hamze *et al*. screened children in Northern Lebanon reporting an RSV incidence of 26.7% with a peak in January 2008 [33], while Finianos *et al*. reported an incidence of 19% with a peak in December 2013 [34]. Globally, detection rates vary tremendously ranging between 4–46% [35]. Therefore, RSV prevalence in this study is in range with those previously reported with a comparable peak activity.

Our findings demonstrate that during the 2016/17 season, two distinct lineages of RSV were co-circulating, ON1 and BA9, with the temporal disappearance of NA2 and BA10 genotypes. Retrospective screening of RSV specimens from the previous seasons, revealed that ON1 and BA9 circulated in Lebanon as early as the 2014/2015 season. The novel LBA2 genotype was detected during the 2014/2015 season but was replaced in the subsequent seasons by the fast-spreading ON1 genotype. Nonetheless, the detection of LBA2 in France suggests a broad geographic spread and warrants further analysis.

The RSV-A ON1 genotype has a 72-nucleotide duplication that was identified initially in Canada and spread worldwide thereafter likely due to the fitness advantage of this genotype [10]. This transition between RSV genotypes in subsequent seasons is believed to be the result of host adaptation, immune selection and evolution of RSV surface proteins [36]. Here, we report a novel genotype named LBA1 that apparently descended from the ON1 genotype. None of the sequences reported in the database belonged to the LBA1 genotype and its geographic spread is yet to be determined.

LBA1 is characterized by six amino acid substitutions and possesses an additional O-glycosylation site (G284S) compared to reported ON1 viruses. While, LBA2, a descendant of NA2 genotype, was characterized by two amino acid substitutions and an additional O-glycosylation site (S292) compared to NA2.

The G glycoprotein is a heavily glycosylated (30–40 O- and 4–5 N-linked glycans) 80 kDa protein with a 289–299 amino acid backbone (32–33 kDa) [37]. The putative receptor binding site is a conserved region of 13 amino acids (164–176) situated within the ectodomain between its two HVRs. Due to their high content of serine and threonine residues, the variable regions are susceptible to extensive post-translational modifications that affect the antigenicity of the G glycoprotein and the overall virus virulence [38, 39]. Post-translational modifications, such as O/N-glycosylation, are also essential to maintain the structural and functional integrity of the G and F proteins [40, 41] whereby endoglycosidase-treated virions displayed significantly reduced viral infectivity [38, 42]. Phenotypic differences of RSV infections were associated with amino acid changes that alter the N- and O-glycosylation patterns [43]. Amino acid substitutions affecting O-glycosylation sites in the G protein were associated with homologous reinfections in children [44]. O-glycosylation was found to be necessary for the binding of most anti-G protein antibodies emphasizing the significance of O-linked carbohydrates for the antigenicity of RSV glycoproteins [42]. Therefore, the addition of glycosylation sites to the Lebanese LBA1 and LBA2 genotypes might be advantageous for the spread of these strains.

Immunocompromised individuals are at greater risk of severe RSV infections. Palivizumab, a high-affinity F-specific humanized murine monoclonal antibody, is effective at preventing the debilitating consequences of the viral infection [18]. The F protein is rich in serine and threonine residues but appears to lack the potential to be O-glycosylated [43]. The F protein is therefore less shielded compared to the G protein and constitutes a more suitable therapeutic target [43]. A concern regarding the increased use of palivizumab is the emergence of antibody-resistant viruses [45]. Clinical isolates with N262D, K272E/M/Q and S275F/L substitutions in the palivizumab-binding site (antigenic site II, amino acids 258–275) exhibited resistance to neutralization by palivizumab [17]. These mutations were linked to breakthrough infections upon prophylactic treatment [46]. Here, we also report that all RSV-A strains circulating in Lebanon carry an N276S mutation that confer partial resistance to palivizumab and constitute a serious concern for the prevention of RSV infections in at-risk population [31, 32]. Our lab recently published the whole genome of an RSV-A ON1 specimen (MG793382) whose F gene also carried N276S [31, 47]. The rate of N276S detection increased over the past seasons even in children without a history of palivizumab treatment [48]. Consistently, N276S mutant viruses have spread in Lebanon despite the infrequent use of palivizumab. The N276S might have originally emerged under antibody-selective pressure, especially that it was nonexistent prior to the use of palivizumab [49]. A number of additional amino acid changes in the F protein (A241V, V247I, Q279H) were also detected among the Lebanese strains whose effect on palivizumab binding is to be determined.

Currently, there is no vaccine available for prevention of RSV infections and candidate vaccines are in various stages of clinical evaluation [15]. It is instrumental to continue monitoring the genetic diversity of the therapeutic targets, to ensure the long-lasting efficacy of anti-viral agents and vaccines.

## Conclusion

In this study, we characterized multiple co-circulating RSV genotypes in Lebanon and identified two novel genotypes, LBA1 and LBA2. Substantial genetic diversity was observed within RSV-A and RSV-B. The increased use of palivizumab along with the universal spread of the N276S mutation represent alarming concerns for the emergence of mutant, resistant viruses and a need for the development of new antiviral drugs and vaccines.

## Supporting information

S1 Fig Predicted PDBsum interface interactions between RSV F protein and palivizumab. **(a)** Predicted interactions within the RSV-A F protein (PDB: 3RRT). **(b)** Predicted interactions within the palivizumab antibody (PDB: 2HWZ). **(c)** Predicted interactions between palivizumab and the RSV-A F protein. **(d)** Predicted interactions between palivizumab and RSV-A F protein carrying the N276S mutation. **(e)** Predicted interactions between palivizumab and RSV-A F protein carrying the N276S and A241V mutations. **(f)** Predicted interactions between palivizumab and RSV-A F protein carrying the N276S and V247I mutations. **(g)** Predicted interactions between palivizumab and the RSV-B F protein. **(h)** Predicted interactions between palivizumab and RSV-B F protein carrying the S276N and Q279H mutations.
(PPTX)
