# Supplementary figures and images for: Detection of ON1 and novel genotypes of human respiratory syncytial virus and emergence of palivizumab resistance in Lebanon
Source: PLoS One. 2019 Feb 21;14(2):e0212687. doi: 10.1371/journal.pone.0212687 (PMC6383889; doi:10.1371/journal.pone.0212687)

## Slide 1
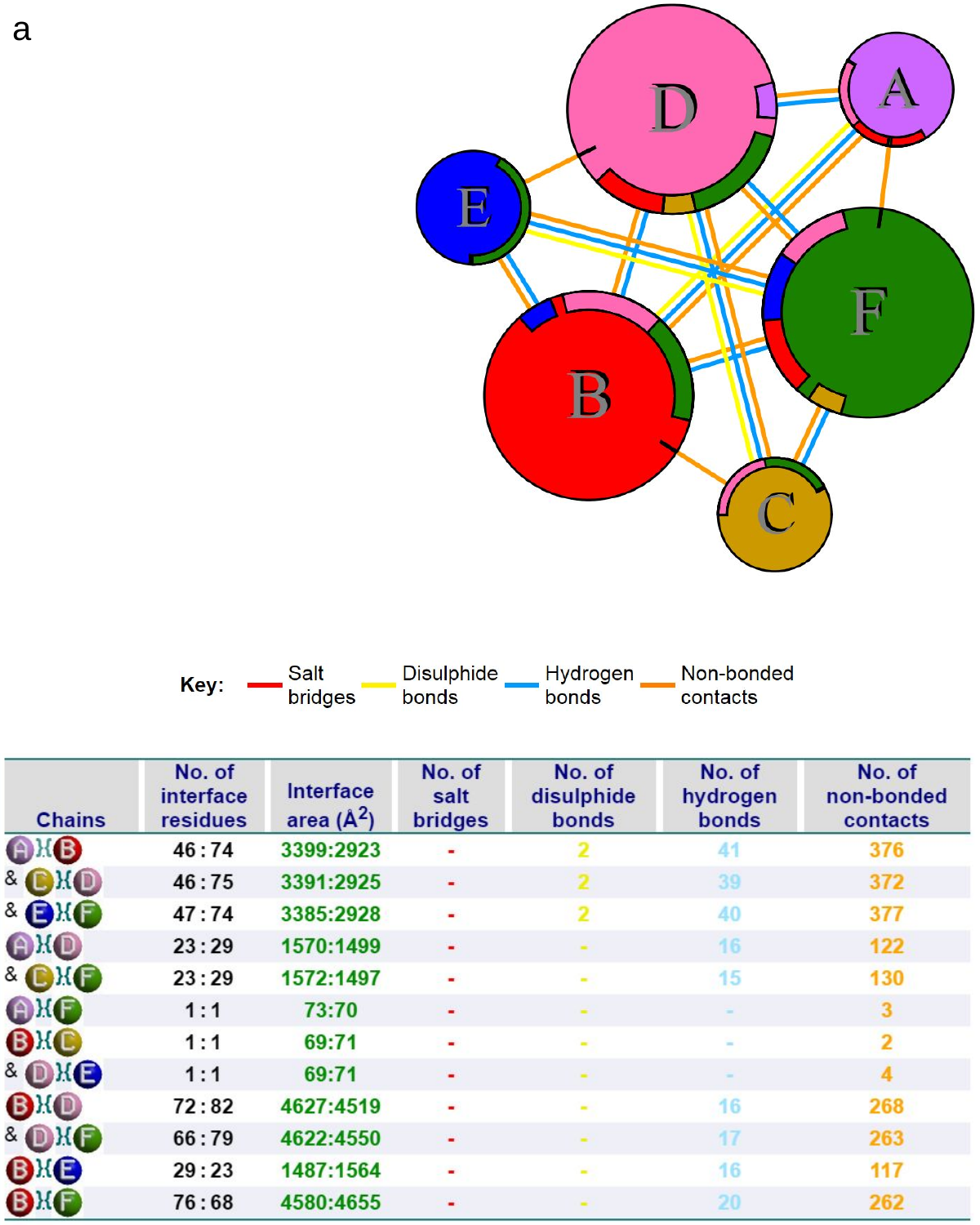

a

## Slide 2
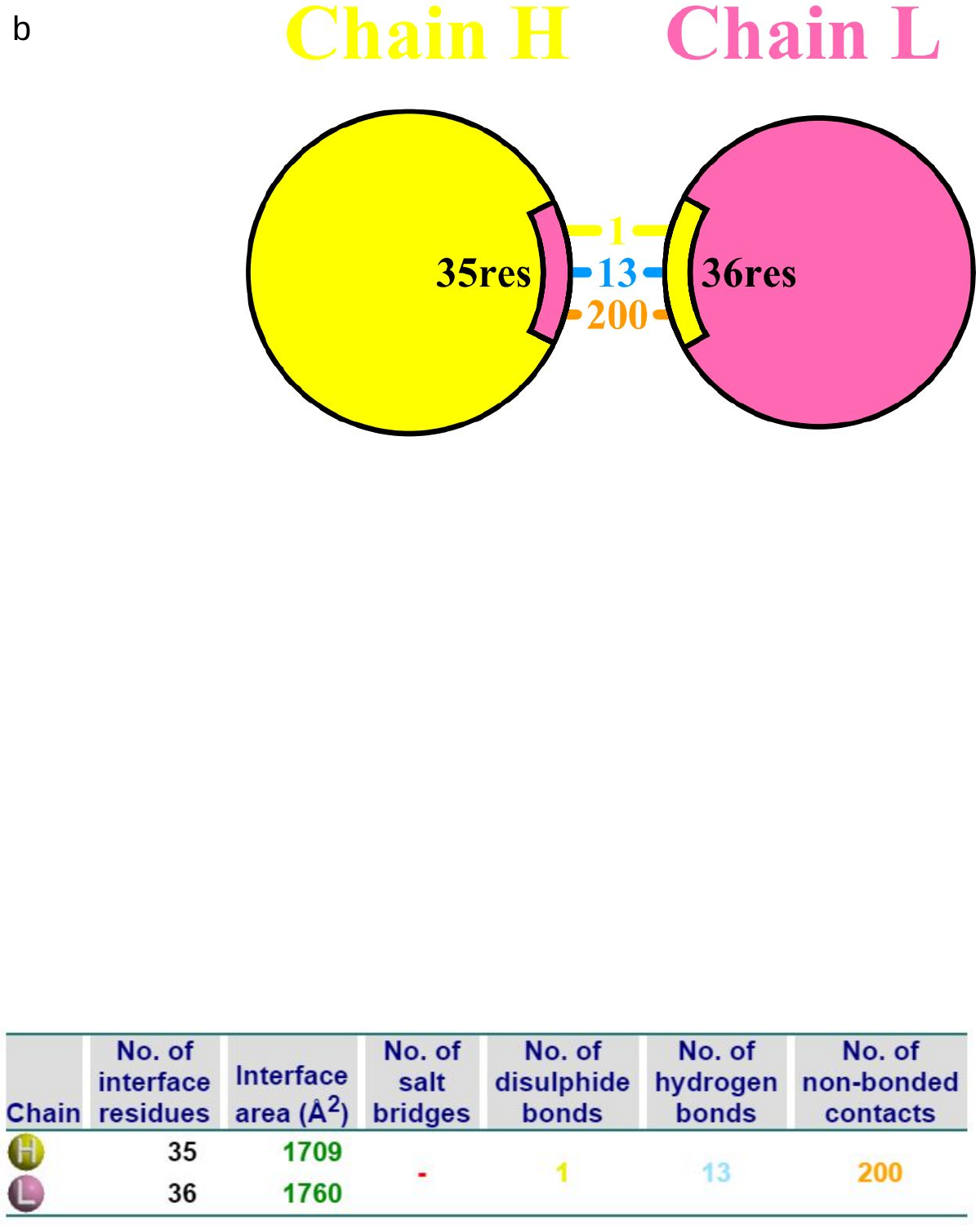

b

## Slide 3
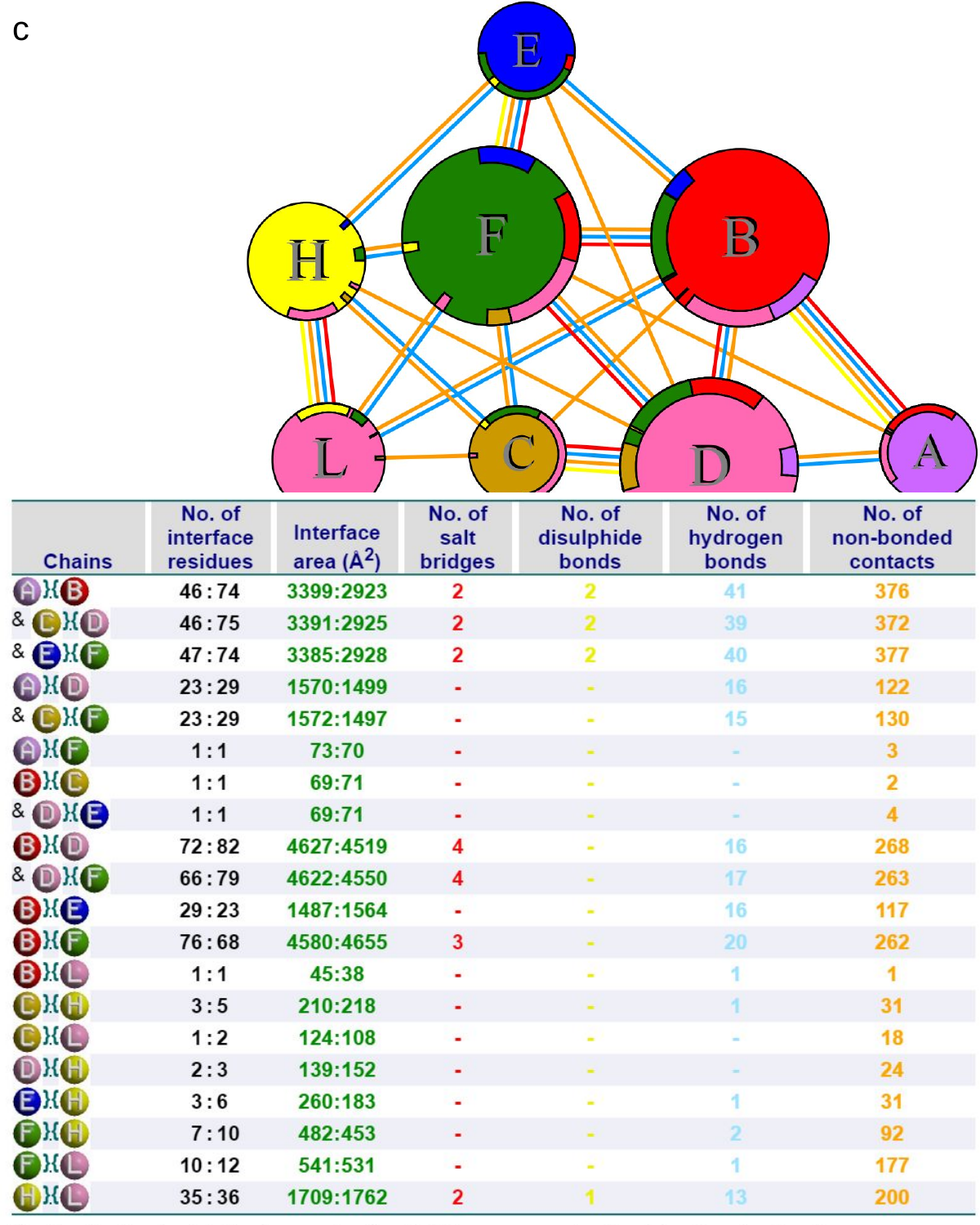

c

## Slide 4
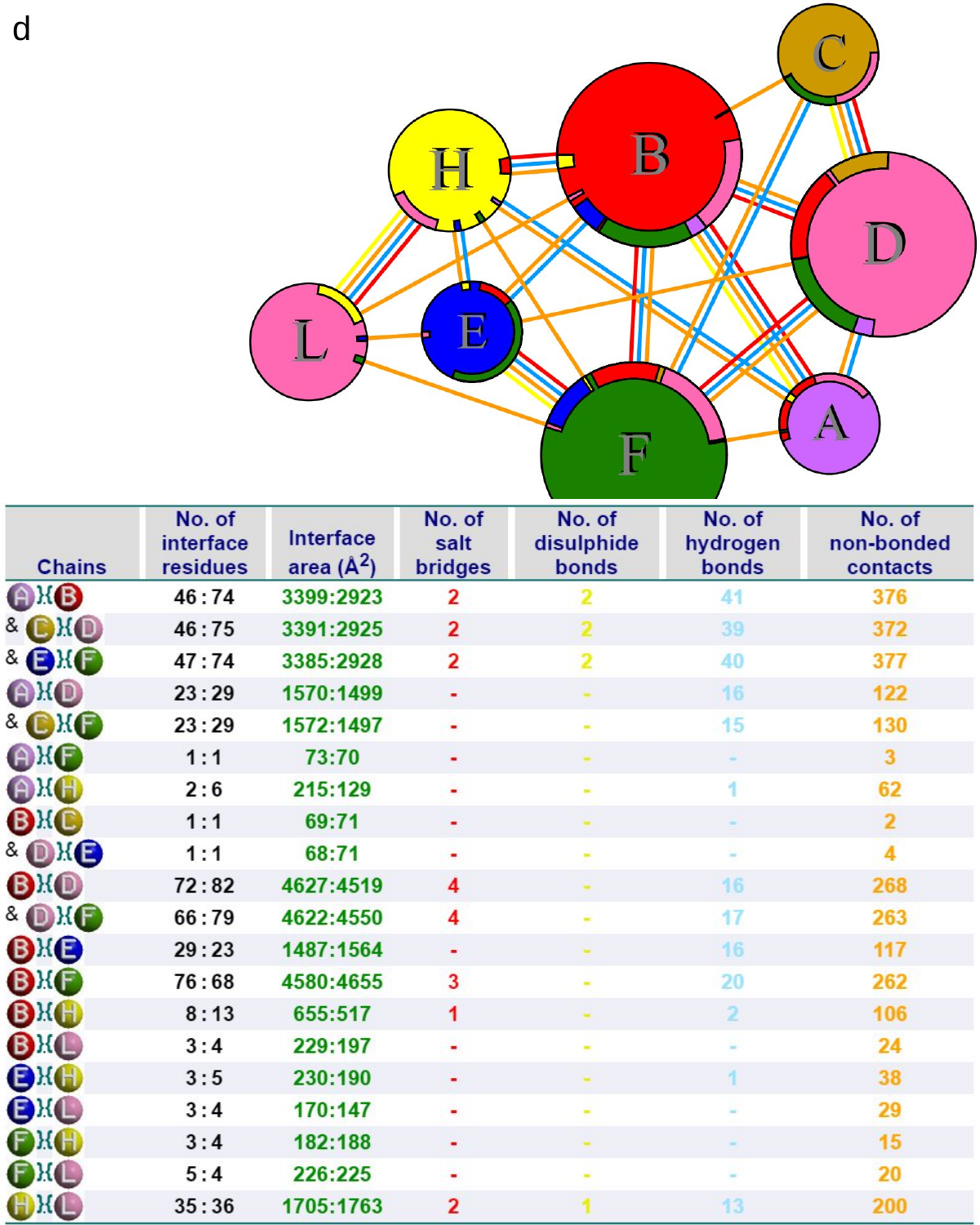

d

## Slide 5
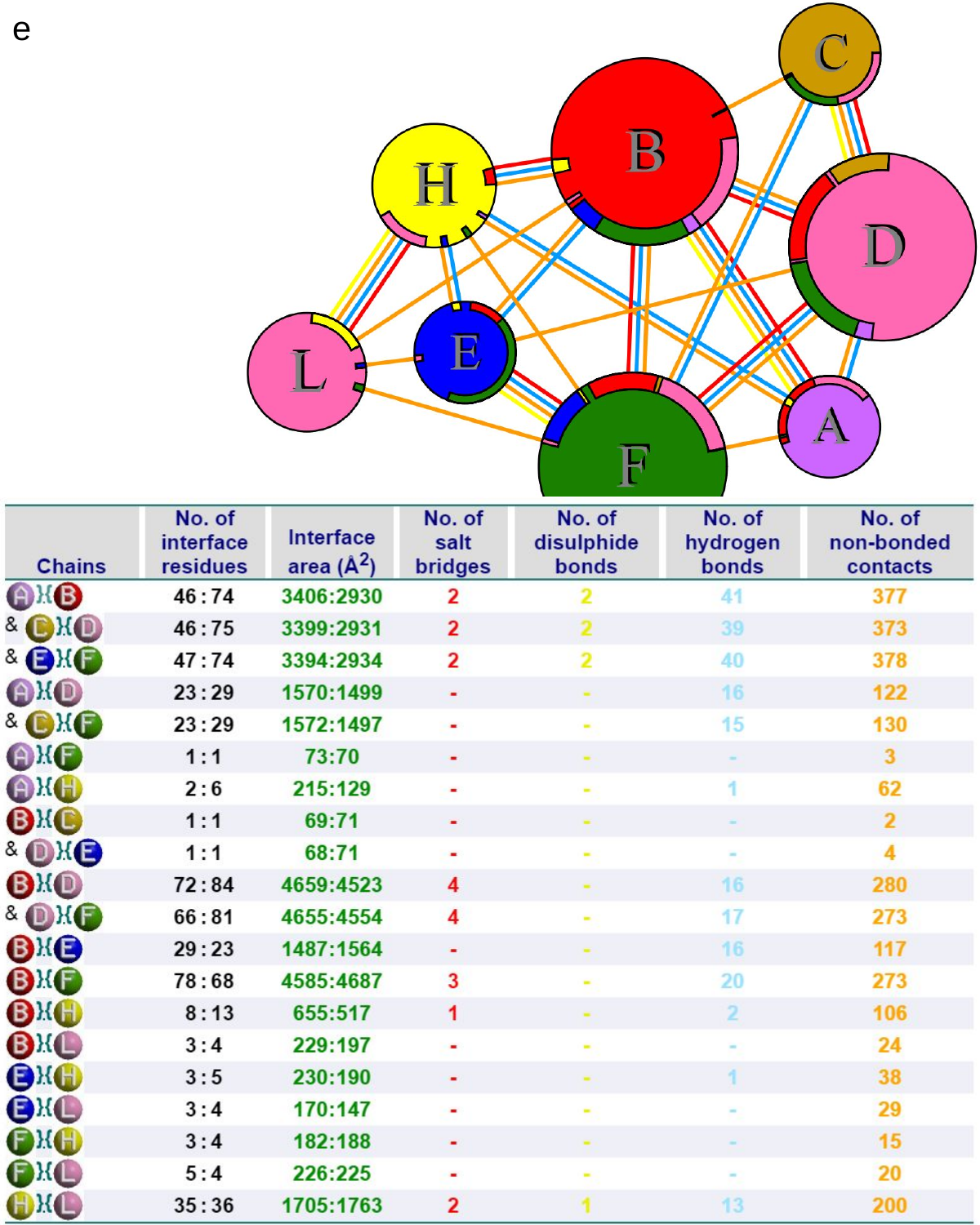

e

## Slide 6
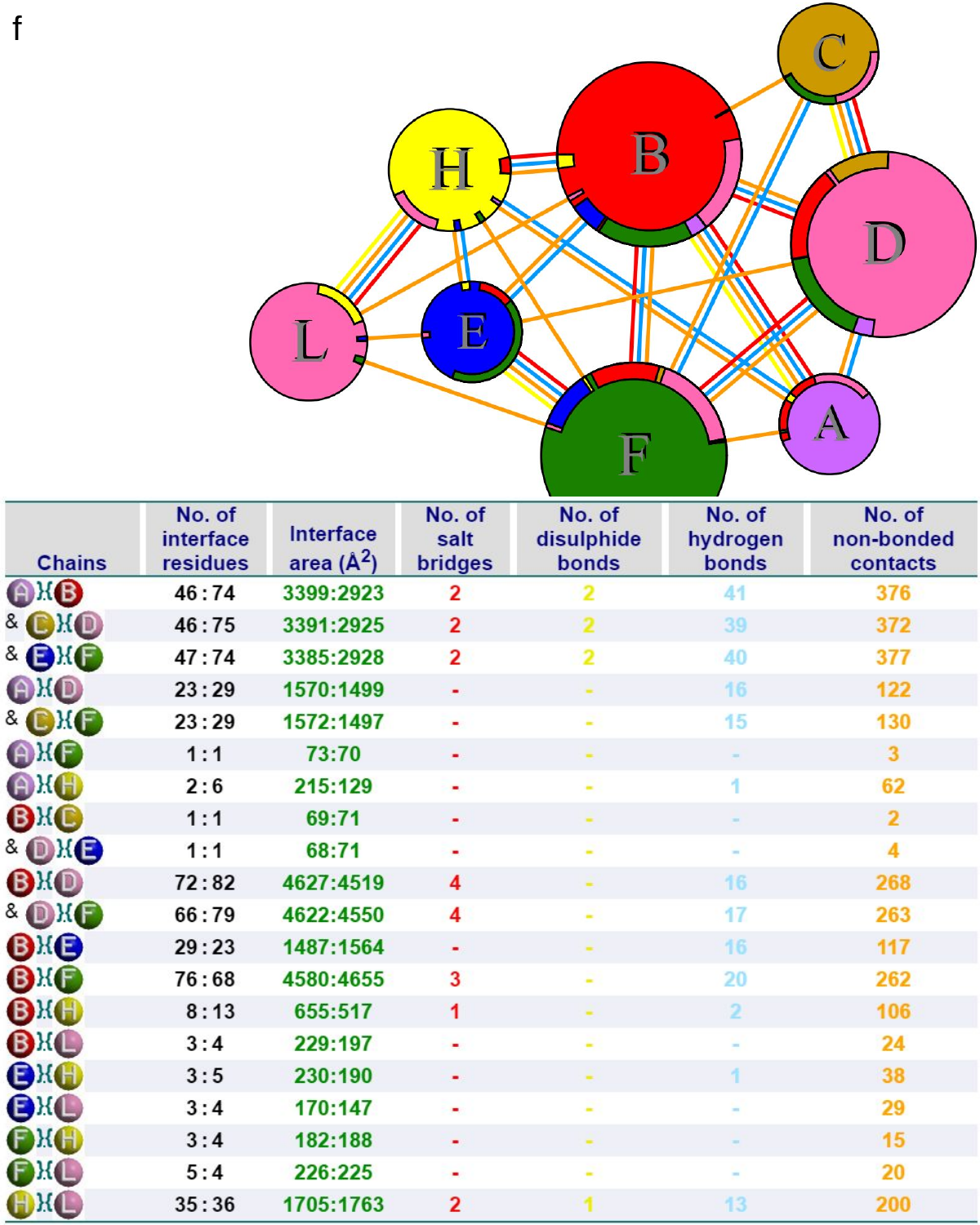

f

## Slide 7
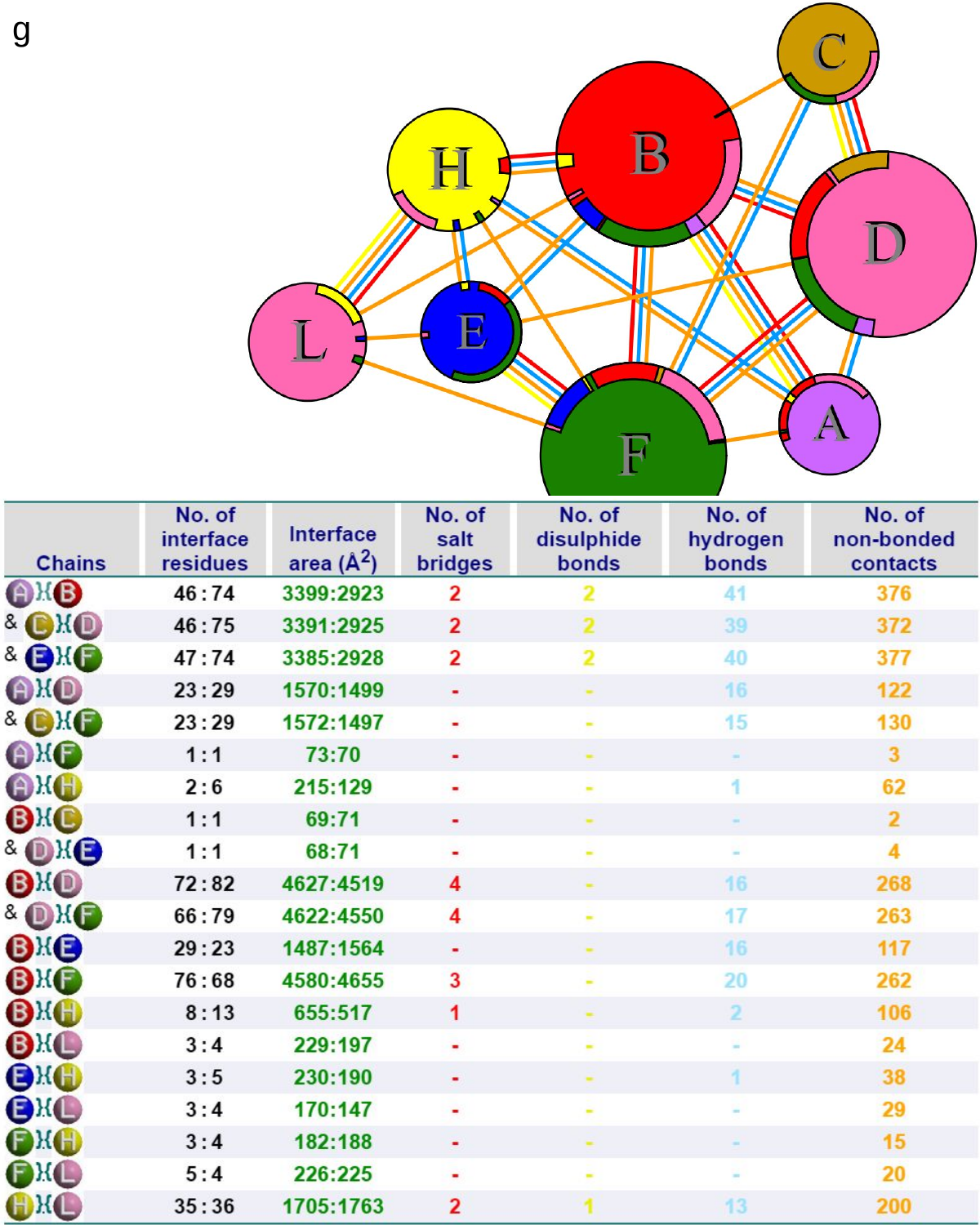

g

## Slide 8
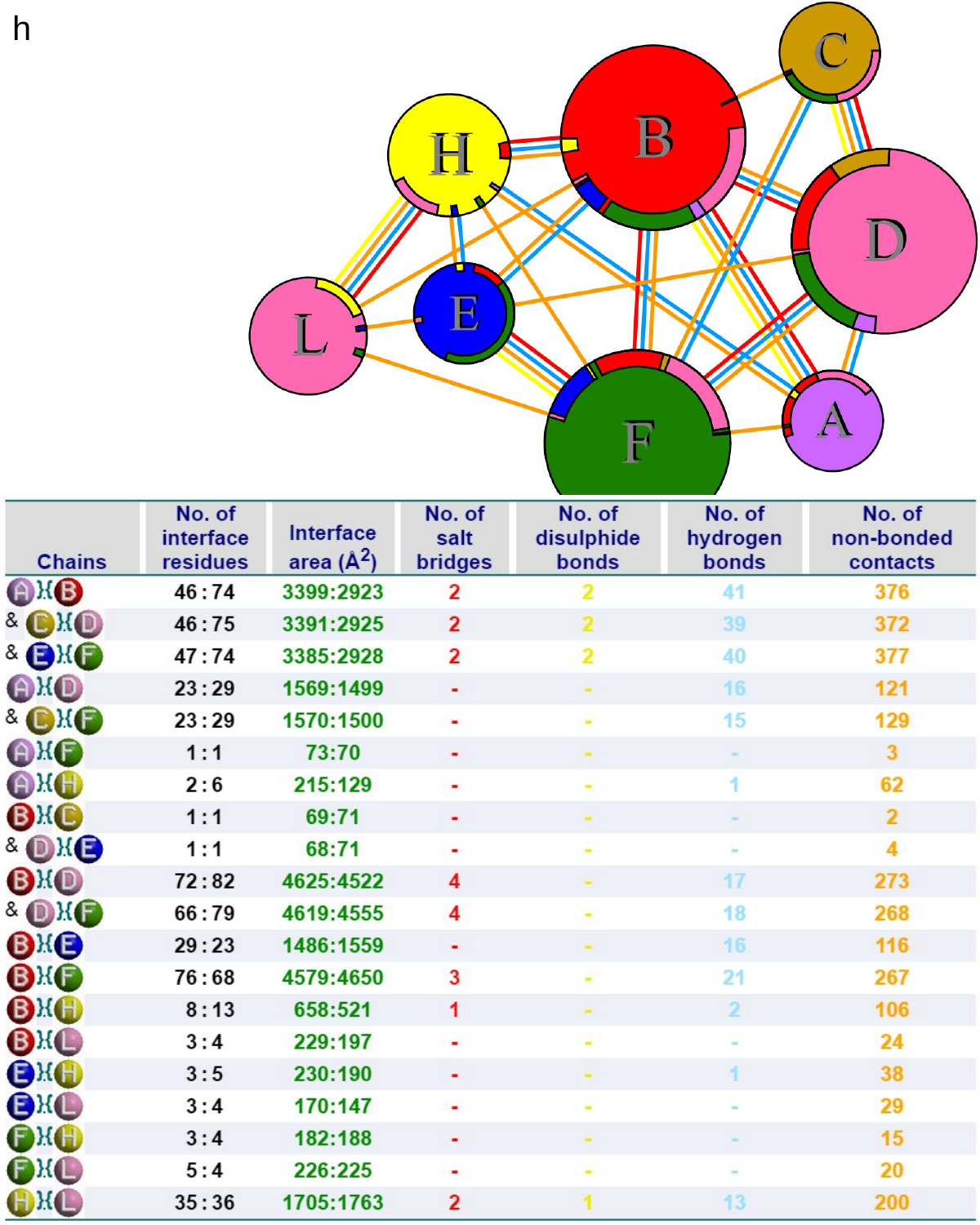

h

Supplement: S1 Fig — (a) Predicted interactions within the RSV-A F protein (PDB: 3RRT). (b) Predicted interactions within the palivizumab antibody (PDB: 2HWZ). (c) Predicted interactions between palivizumab and the RSV-A F protein. (d) Predicted interactions between palivizumab and RSV-A F protein carrying the N276S mutation. (e) Predicted interactions between palivizumab and RSV-A F protein carrying the N276S and A241V mutations. (f) Predicted interactions between palivizumab and RSV-A F protein carrying the N276S and V247I mutations. (g) Predicted interactions between palivizumab and the RSV-B F protein. (h) Predicted interactions between palivizumab and RSV-B F protein carrying the S276N and Q279H mutations. (PPTX) [file pone.0212687.s001.pptx]
